# Extrusion-Based 3D Printing of Microfluidic Devices for Chemical and Biomedical Applications: A Topical Review

**DOI:** 10.3390/mi9080374

**Published:** 2018-07-27

**Authors:** Daniela Pranzo, Piero Larizza, Daniel Filippini, Gianluca Percoco

**Affiliations:** 1 Masmec Biomed, Masmec S.p.A. Division, 70026 Modugno (Bari), Italy; daniela.pranzo@masmec.com (D.P.); piero.larizza@masmec.com (P.L.); 2 Optical Devices Lab, IFM, Linköping University, 58183 Linköping, Sweden; danfi@ifm.liu.se; 3 Department of Mechanics, Mathematics and Management, Polytechnic University of Bari, 70126 Bari, Italy

**Keywords:** FFF, FDM, 3D printing, biomedical devices, chemical reactors, microfluidics, lab-on-a-chip (LOC)

## Abstract

One of the most widespread additive manufacturing (AM) technologies is fused deposition modelling (FDM), also known as fused filament fabrication (FFF) or extrusion-based AM. The main reasons for its success are low costs, very simple machine structure, and a wide variety of available materials. However, one of the main limitations of the process is its accuracy and finishing. In spite of this, FDM is finding more and more applications, including in the world of micro-components. In this world, one of the most interesting topics is represented by microfluidic reactors for chemical and biomedical applications. The present review focusses on this research topic from a process point of view, describing at first the platforms and materials and then deepening the most relevant applications.

## 1. Introduction

Extrusion-based additive manufacturing (AM) was developed in 1988 by S. Scott Crump and commercialized by his company, Stratasys, under the name fused deposition modelling (FDM). The process is often referred to as fused filament fabrication (FFF).

**Figure 1:**
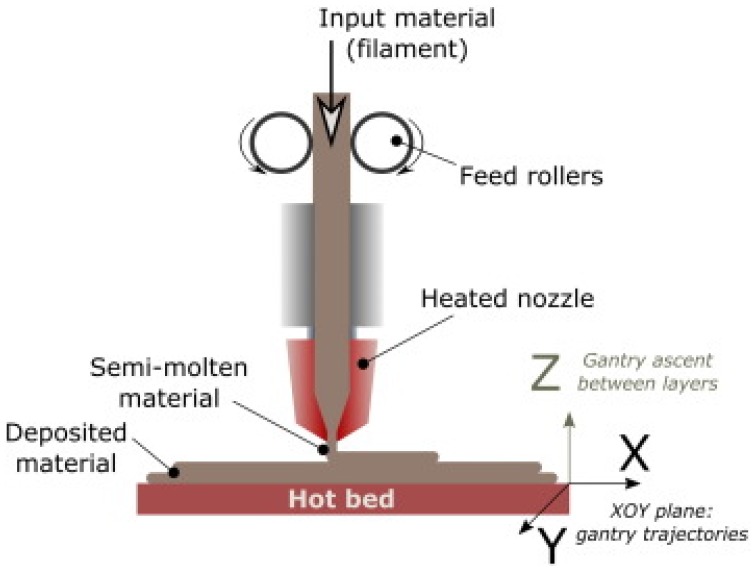
Fused filament fabrication (FFF) process (reproduced with permission from [1]).

Generally, FFF-based printers use a thermoplastic filament that is unrolled from a spool such that the material is pushed toward an extrusion head (including one or more extrusion nozzles) and toward drive wheels, which are necessary to control the flow. The nozzle is heated to semi-cast the material, and the head can be driven both horizontally and vertically by a numerical control mechanism, following a path traced by software (Figure 1).

Thanks to the expiration of the patent and the consequent large open-source community development, many commercial variants arose, making FFF one of the most commonly used AM processes. Its advantages include low purchase and maintenance costs [2], a wide choice of commercially available materials, easily changeable materials, nontoxic materials, compact platforms, and low-temperature operation. These benefits make FFF a very popular technology, well-suited for microengineering, continuously evolving, and overcoming the limited capabilities for small parts manufacturing that characterized this technology just few years ago [3]. However, their main disadvantages are surface roughness, imperfect sealing between layers and toolpaths, need for support material, support removal, and long building times for massive pieces. FFF can be used to extrude metallic materials, hydrogels, or cell-loaded suspensions in order to incorporate functional components (such as sensors, actuators, batteries, strain sensors, antennas, interconnects, and electrodes) in microfluidic devices [4]. Moreover, unlike traditional microfluidic manufacturing methods (i.e., soft lithography) that require specialized fabrication skills and facilities, FFF is accessible and customizable to serve biology, chemistry, or pharma research and development needs [5]. Furthermore, open-source technologies enable researchers to improve the design process and reduce production for specific applications [6].

In this context, biomedical and chemical microfluidic applications represent one of the most studied topics.

Other interesting review papers dedicated to the topic of additive manufactured lab-on-a-chip (LOCs) are reference [7], where the authors discuss a broad range of approaches for the application of 3D printing technology to fabrication of micro-scale lab-on-a-chip devices; reference [8], where the most recent trends in 3D-printed microfluidic devices are discussed, with a focus to the fabrication aspects of these devices, including a microfluidic channel, threads to accommodate commercial fluidic fittings, a flow splitter, a well plate, a mold for polydimethylsiloxane (PDMS) channel casting, and how to combine multiple designs into a single device; reference [9], where it is stated that 3D printing can aid the field of microfluidics in finding its “killer application” and a review is carried out of how 3D printing helps to improve the fabrication of microfluidic devices; reference [10], a critical review that focuses on inkjet (i3DP), stereolithography (SLA), two photon polymerisation (2PP), and extrusion printing; and reference [11], which foresees widespread utilization of 3D printing for future developments in microfluidic engineering and lab-on-a-chip technology.

However, these papers are mainly focused on photopolymerisation-based additive processes. Nevertheless, there is emerging evidence that extrusion-based processes can gain much importance in microfluidic applications, owing to their inherent simplicity and versatility to accommodate well-defined materials, as well as their continuously evolving performance. In fact, the exploitability of the process is certified by a protocol [12], available in the literature, for the 3D printing of versatile reactors for chemical synthesis that describes two different approaches to FFF fabrication. Accordingly, this article reviews the ongoing efforts in FFF for chemical and biomedical microfluidics applications. With respect to millifluidics with FFF, please refer to [13] for guidelines on connection concepts, gas tightness, and probe insertions, as well as its outlook.

The present review paper at first focusses on FFF machines and exploited materials for microfluidics, and then more deeply considers the most relevant applications. The remainder of the paper is organized as follows: Section 2 discusses FFF materials and post-treatments, Section 3 describes FFF machines for microfluidics, including costs, Section 4 discusses applications, and a comparison with photopolimerization processes in Section 5. Bioprinting is not considered in this paper. For this topic, please refer to [14].

## 2. Materials and Post-Treatments

Although FFF might appear topologically ill-suited for producing microfluidic devices [4], because of possible leaks due to poor sealing between the single beads, it has recently gained diffusion in microfluidics, thanks to the possibility of tuning the process parameters and of increasing the positioning accuracy, as well as to the reduction in available nozzle diameters.

The main microchannels fabrication challenges using the FFF process are [15]

- The extruded filaments cannot be arbitrarily joined at channel intersections;
- the seals are weak owing to the lack of structural integrity between the layers;
- the size of the extruded filaments can be larger than channel sizes used in microfluidics.

The existing approaches for the fabrication of microfluidic devices, described in [16], for 3D printing in general are also applicable for FFF.

- AM of templates for replicas of conventional materials, such as polydimethylsiloxane (PDMS) or poly(methyl methacrylate) (PMMA); this fabrication technique is also referred to as rapid tooling in the manufacturing community.
- Direct AM of microchips, including both open channel to be sealed and closed channel.

### 2.1. 3D Printed Materials for Microfluidics

A notable advantage of FFF is that it can process a large variety of thermoplastic polymers. Because thermoplastics are used for mass fabrication such as hot embossing or injection moulding, FFF devices are made from materials that are the same as those used in mass production techniques. The most common materials are acrylonitrile butadiene styrene (ABS), polystyrene (PS), and polycarbonate (PC), as well as biocompatible polymers such as polycaprolactone (PCL), polylactic acid (PLA), polybutylene terephthalate (PBT), and polyglycolic acid (PGA).

FFF machines have undergone rapid dissemination after the expiration of the Stratasys patent. Such popularity has been supported by a significant reduction in cost and launch of new extrudable materials. The improvement of the process has not been so fast and actually the most representative specifications of an FFF 3D printer are the build size and layer thickness, sometimes referred as “resolution.” Currently, maximum available build sizes are in the order of 300 L (for example, a cylinder with diameter 600 mm and height 1000 mm of the DELTA WASP 60,100 w), and minimum layer thicknesses are approximately 10 µm, declared by Leapfrog A0275 Xeed 2.0. This type of specification is thus not comparable with other AM technologies, which employ a stricter resolution based on voxel (volumetric pixel) size and raw surface finish.

With respect to extruded materials for microfluidics, they are essentially polypropylene (PP), ABS, and PLA.

PP is used essentially for its high biocompatibility; in this way, it is very similar to PDMS but cheaper. As a consequence, it is claimed to be an attractive material for the additive fabrication of micro- and milliscale microfluidic devices, being a robust, flexible, and chemically inert polymer [17,18,19]. However, PP is not widely used in the FFF community. In fact, PP is a semicrystalline material: it does not gradually soften with increasing temperature and rapidly changes into a low-viscosity liquid, shrinking less in the flow direction than in the transverse direction. As a consequence, PP is in the liquid state when extruded and, when cooling, the crystallization starts as soon as the temperature drops below the melting point. Thermal contraction stresses are high during the solidification of layers, resulting in very high warping stresses. Consequently, PP should be used only when high biocompatibility is required.

On the contrary, as an amorphous polymer, ABS is able to slowly creep until it cools below the glass point, partially compensating thermal stresses above this temperature and starting to warp below the glass point, to full solidification. In this context, heated build plates or chambers are useful to minimize warping stress, with a temperature set around the glass point.

While ABS is not widely used in medical and microfluidic devices, in comparison to materials such as PDMS, its superior mechanical and processing properties, versatility, and low cost can make it a very useful material for several biomedical applications. However, the layers tend not to blend together, with small gaps and holes between the deposited material. The process parameters must be accurately tuned to avoid this limitation, or a chemical treatment must be performed (see below). Moreover, biocompatibility can be improved minimizing protein and other biomolecule adhesion during flow, for example through the grafting of poly (ethylene glycol) [20]. 

More recently, PLA has replaced ABS as the most common material for 3D printing. Some advantages of PLA are that it is derived from renewable resources, biodegradable, nontoxic, and inexpensive. PLA belongs to the family of aliphatic polyesters. The monomer can be easily cured with good yield, high molecular weight, and amorphous or semicrystalline polymer characteristics depending on the percentage of the two L and D stereoisomers. Consequently, for 3D printing filaments, it is manufactured to be amorphous, which is more affine to FFF. Moreover, users often experience lower sensitivity to moisture and lower tendency to obstruct the nozzle. Its recent use [21,22,23] in microfluidic devices has mainly been due to its diffusion into the AM community.

### 2.2. Post-Treatments

Another interesting aspect is represented by post-treatments, as the importance of post-treatments in AM processes is well-known. Notwithstanding this, in several examined papers, no treatments are described at all. The application of post-treatments to fabricated devices is mainly related to (i) reduction of fluid leakage and (ii) surface functionalization.

With respect to (i), fluid leakage can be: (a) between beads; (b) at fluidic connections; and (c) in correspondence with sealing tape, when the tape is necessary.

With regard to leakage between beads (a), the most common method is based on solvents that weld the threads after evaporation or reduction of vapor solvent concentration [20,21]. Despite its appeal to overcome the practical limitation of FFF structural problems, chemical polishing has collateral effects. The most prominent of these is the erosion of geometric features, which can be critical in complex architectures. Accordingly, it is certainly a more attractive proposition to secure structural integrity and entirely avoid post-treatments by the specialized control of printing parameters. With respect to (b) leakage on connection, an example of treatment is given in [24], where, because a plug and play approach is proposed for fluidic devices, this topic is very critical. In that case, Dichloromethane (DCM) vapor is applied for up to 15 min to smoothen the connectors and avoid leakage. Regarding (c), the top surfaces of open 2D channels are sealed with a PP adhesive tape. This tape can efficiently seal the top surface of the device only if this surface has a sufficiently low roughness. This quality can be easily attained with most materials by simply using sandpaper [25]; another interesting approach, applied to PLA devices, is given in [22], where an open channel PLA device is sandwiched between glass elements and “annealed” to 170 °C to decrease both the thickness of the PLA spacer and the width of the channels, as well as to smoothen the faces of the device, thereby achieving a liquid-tight seal.

From a process point of view, type (a) and (c) leakages are avoidable with FFF only if particular attention is paid to the process parameters. In fact, process parameters can be set up to increase the superimposition between the beads, reducing the leakage possibility; moreover the “ironing” function can be used to avoid surface treatments for sealing tapes. The ironing function consists in passing the hot nozzle on the top slice, without extruding material.

The second issue related to post-treatments is linked to the functionalization of surfaces. Examples are given in [26,27] in which PP is coated with silver paint after thoroughly cleaning the device in detergent solution with ultrasound. Then, the paint is cured at 120 °C. The post-treatment in this case is aimed to make the device suitably conductive for electrocoating.

## 3. FFF Machines for Microfluidics

This chapter will focus on the FFF-based machines used in literature for microfluidic applications. Depending on their intended use, they can be classified as follows:

- Consumer grade: their price is generally lower than $1000, and they are normally directed to home and personal use, focusing more on lowering costs than on enhancing reliability and accuracy. Some of them, such as RepRap, must be assembled by the user.
- Professional grade: their price is typically between $1000 and $10,000; the layer thickness can reach 10 µm, but the build volume is in the low-to-medium size range (up to 84 L for the DELTA WASP 4070 PRO). Reliability and processing speed are not crucial specifications, but their performance tends to follow the price.
- Industrial grade: costlier than $10,000; the main builder is Stratasys. This market segment is characterized by addressing rapid manufacturing needs, which is the production paradigm that considers manufacturing with zero machining setup times. Reliability and speed are essential.
- Customized or self-built machines: normally consumer-grade machines that have been modified by researchers to achieve specific features, such as increased nozzle temperature and reduced nozzle diameter.
- Dedicated FFF machines.

### 3.1. Consumer-Grade FFF Machines Employed in Microfluidics

Fab@Home [28] was the first multimaterial 3D printer for consumers, and together with the RepRap project, was part of the first two low-cost and “do it yourself” 3D printers, whereas their predecessors were industrial grade platforms. RepRap was self-defined as “humanity’s first general-purpose self-replicating manufacturing machine” and takes the form of a desktop 3D printer capable of printing plastic objects. Because many parts of RepRap are made from plastic and RepRap can print those parts, the platform can self-replicate by making a kit of itself, and anyone can assemble it, given enough time and materials [29].

The Fab@Home project ended in 2012, when it was clear that the project goals had been achieved, whereas RepRap is still developing and improving low-cost AM machines such as the Prusa and Darwin models.

The Fab@Home platform was used for reactionware using two steps: (i) the device is additively manufactured at first using a robust, quick-curing acetoxy silicone polymer (Loctite 5366 bathroom sealant, LOCTITE) then inserting non-printable components (glass frit and microscope slide/indium tin oxide (ITO) viewing window) during pauses [30]. No dimensional measurements were performed in this work. Two fabrication steps were also used with Fab@Home for the fabrication of a microfluidic reactors [31]. In this case, a Fab@Home Version 0.24 RC6 freeform fabricator and a Bits from Bytes 3D Touch 3D printer were employed. Bytes 3DTouch was later incorporated by 3D Systems, and finally discontinued. These two different extrusion machines enabled configuring a propylene base with the 3D Touch machine, which provided an inert material for the solvents and reagents necessary for the organic transformations. The device was then completed in the Fab@Home machine, where the catalyst components were deposited in the hosting architecture. The catalysts were delivered within an acetoxy silicone polymer matrix to allow the material to be extruded by the Fab@Home printer. The device was then finished in the 3D Touch machine. This switch between printers is arguably a source of manufacturing errors and demands alignment. Such problems could be avoided by using a single, multi-nozzle extruding machine.

### 3.2. Professional-Grade FFF Machines Employed in Microfluidics

One of the most used professional 3D printers is the 3D Touch from Bits for Bytes. Its production was discontinued in 2013 after 3D Systems absorbed Bits for Bytes in 2010. It can print several plastic materials and can reach a layer height of up to 125 µm, lateral tolerance in the x and y axes ±1% of object dimension or ±0.2 mm (0.008″/200 µm), build volume 27.5 × 27.5 × 21 cm^3^, minimum layer height 125 µm, up to three extruders, and printing speed 15 mm^3^/s.

Some examples of microfluidic applications of this machine are semicircular channels fabricated in PP, with diameters ranging from 0.8 to 1.5 mm, in [17,18,32], and rectangular channels [26] with cross-sectional area of 0.9 × 0.9 mm^2^.

An additional widespread professional 3D printer used for microfluidic applications is the Ultimaker 2 (Ultimaker, Geldermalsen, The Netherlands). This machine can build in ABS, PLA, and exotic materials, has a heated base, minimum layer thickness 20 µm, and declared XY precision of 12.5 µm. Some examples of microfluidic applications have been discussed [27], as well as the modular architecture of PLA and polyethylene terephthalate (PET) parts [24]. In the former, circular channels of diameter 0.8 mm were realized, and in the latter, a droplet generator device with the same channel size was designed.

The Makerbot replicator 2X is one of the FFF machines with the poorest resolutions and is characterized by a minimum layer height equal to 100 µm, two extruder heads, XY precision declared equal to 0.011 mm, and a heated platform. Some examples of applications are [33] building fluidic devices for nanoparticle preparation and electrochemical sensing (channels with square cross-section of 800 × 800 µm^2^), exploiting the FFF capability of extruding more than one material on the same slice, and in ([23]), where a microfluidic immunoarray was printed from PLA (reagent chambers volume of 170 µL).

Profi3DMaker, with a printing volume of 400 × 260 × 190 mm^3^ and z resolution of 0.08–0.25 mm, employed in [34] and in [35], was used for bacterial cultivation, lysis, DNA isolation, PCR, and the detection of methicillin-resistant Staphylococcus aureus.

Other frequently employed professional 3D printers include: HD2x from Airwolf3D [22], (used to create one layer (200–250 µm) mixers in PLA), the Leapfrog Creator [36] for the rapid fabrication of cyclonic spray chambers for inductively-coupled-plasma-based techniques, and the miniFactory 3 by miniFactory Oy Ltd. (Seinäjoki, Finland), equipped with a 0.4-mm diameter nozzle, to fabricate a miniaturized PP reactor.

### 3.3. Industrial-Grade FFF Machines Employed in Microfluidics

The high costs of industrial AM machines restrict them to specialized infrastructure, and they were more used in at the beginning of 3D printing development in the early 2000s. The Stratasys FFF 3000 system, using ABS and layer a thickness 0.178 mm, to fabricate channels as small as 500 µm in width and depth is an example of these early developments [37]. At that time, strong limitations were found, mainly due to the inaccuracy of the machine and air gaps between the roads defined by the toolpaths. In addition, such platforms lacked the affordability currently associated with FFF printers.

The Dimension SST 768 3D Printer (Stratasys, Inc., Eden Prairie, MN, USA), employing 254 µm slices of ABS with channel widths of 518 µm (meaning that only two slices were involved in these features) was studied by [25]. Sanding of the surface was used to improve sealing with commercial PP tape. The use of high height slices probably reduced the leakage between solidified filaments, but internal leakage could not be eliminated.

A Stratasys Dimension was used also in the demonstration of a method to modify the surfaces of ABS with microstructured features, which render them water-impermeable, hydrophilic, and biocompatible [20]. The treatment utilizes an acetone-based sealing method, described as minimally impacting on surface roughness and structural fidelity; this was followed by photo-induced graft polymerisation of polyethyleneglycol onto the surface, which increases the hydrophilicity of the ABS and its resistance to non-specific protein binding.

### 3.4. Customised Customer Grade

The redesign of the extruder in a Zhejiang Flashforge 3D platform has been demonstrated with the purpose to melt sugar for pneumatic extrusion and dispensing [38]. This machine was based on the open-source RepRap Megatronics motherboard, and the extruder incorporated a heating device to melt the sugar and also to maintain a steady extruder temperature. The heater consisted of a thermistor temperature sensor and custom-made polyimide electrothermal membrane (100 × 100 mm^2^, 12 V, 90 W). The enclosure of the sugar extruder was surrounded by the electrothermal membrane, and the entire sugar extruder was covered with polyimide tape for thermal insulation purposes; the motherboard acted as a proportion integration differentiation (PID) controller to retain steady temperatures of ±0.5 °C. Replaceable nozzles were utilized for the sugar and PDMS extruders to prevent blocking by solidification, simplify parts replacement, and improve cost efficiency.

### 3.5. Dedicated FFF Machines

An FFF machine called Fluidic Factory has been recently launched to the market by Dolomite Microfluidics, as the first machine dedicated to creating sealed 3D microfluidic devices. The only available material is cyclic olefin copolymer (COC), a solvent-resistant, hard, transparent, and medical-grade plastic. The declared resolution limit of the COC layers is 320 µm (w) × 125 µm (h), but the z resolution, i.e., the height of the layer, is not clear. The microfluidic devices are printed through a 60 µL volume of COC polymer, melted to a fluid state held a few seconds before being ejected and deposited in a ‘squashed’ manner, ensuring adherence and allowing filaments to melt together to generate leak-free channels. The price is in the range of industrial printers and it can only produce translucent, leak-free, closed, and impermeable microchannels [39].

### 3.6. FFF Machines Comparison

In Table 1, the applications are summarized together with the machine and material employed, its specifications, and post-treatments necessary for the application. The parameter chosen for a dimensional comparison is the size of the microchannels, which can have rectangular or circular shape. In case of a circular shape, the value considered is the diameter of the channel, indicated by D in the table, whereas in the case of semicircular channels, R indicates the radius of the channel. The motivation to avoid comparison based on printer specifications is that they are not strictly defined across different technologies.

The only machine feature considered in the comparison is the resolution in the z axis, which corresponds to the slice height and is equivalently defined across printing technologies. The table has been arranged based on the machine and then publication time.

### 3.7. Resolution

Table 1 shows that the minimum microchannel size fabricated with FFF is 200 μm, described in [38], indirect method and [22], direct method. The former exploits the Creator pro 3D printer by Flash forge^TM^, with a declared layer resolution of 100–500 µm and positioning precision on XY: 11 µm and Z: 2.5 µm. The latter exploits an Ultimaker 2 with the following specifications: layer resolution 20–200 µm and XYZ positioning accuracy of 12.5, 12.5, and 5 µm.

In several cases, it can be noticed that 3D printers are sometimes exploited improperly, achieving depth of channels that only double the minimum slice height, thus including only two beads in height for those channels and leading to a very low channel quality.

## 4. FFF Microfluidic Applications

**Figure 2:**
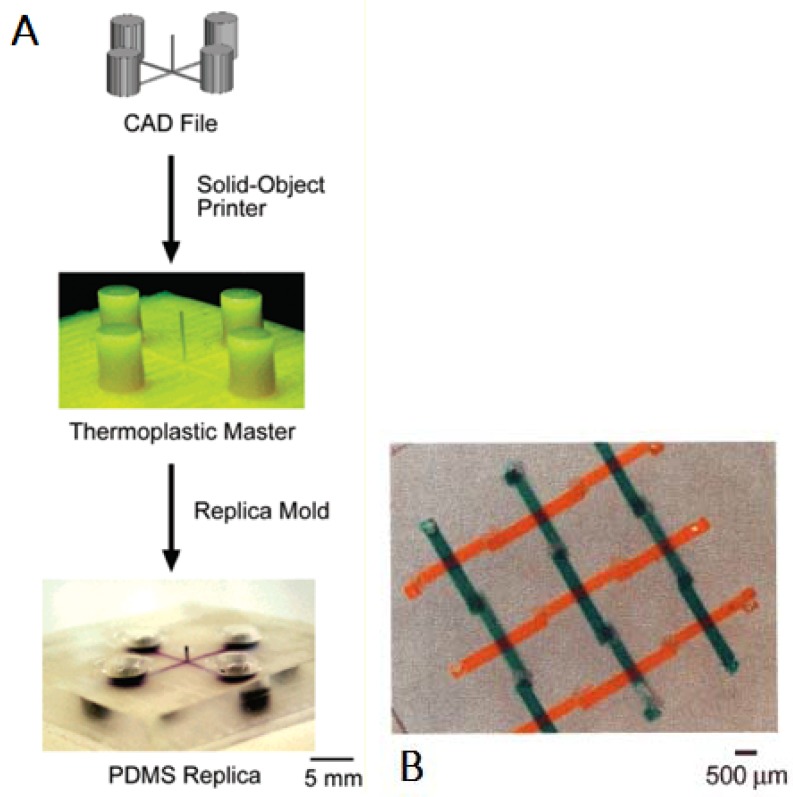
(**A**) Scheme for prototyping devices in polydimethylsiloxane (PDMS) using solid-object printing; (**B**) Basket weave pattern for crossing, nonintersecting channels. (Reproduced with permission from [40].)

As mentioned in [9], the first applications to microfluidic devices were in the early 2000s: one of the first examples related to FFF is shown in Figure 2 [40], where an FFF machine is used for rapid tooling to manufacture microfluidic devices in PDMS, with a reported 250 µm resolution. Since this initial result, the variety of extrudable materials has notably increased, leading to increasing opportunities for research.

FFF has also been used to create channel volumes as a sacrificial material. This approach is limited to circular channel cross-sections, without internal features, and to special types of channel junctions, composed of orthogonal cross-overs [4].

Generally speaking, the devices reported in research can be classified into three categories.

- Devices based on 2D open channels;
- Devices based on 2D closed channels;
- Devices based on 3D geometry.

In the first category, the 3D printer is used to build channels with an open top that is subsequently sealed with bonded glass or a special adhesive tape. This choice is normally chosen when channel observation must be conducted, minimizing support deposition and manufacturing defects.

In the second category, the 3D printer is used to build channels that are not designed to be observed but just to carry fluids. Manufacturing defects are more probable with this configuration, owing to a higher building complexity.

These two categories are studied to transfer the manufacturing features, normally developed with time-consuming and high-cost lithographic techniques, to fast and cheap additive processes.

The third category tries to exploit the real potential of additive processes in fabricating hollow 3D structures, which are very hard to fabricate with more conventional processes.

### 4.1. Devices Based on 2D Open Channels

**Figure 3:**
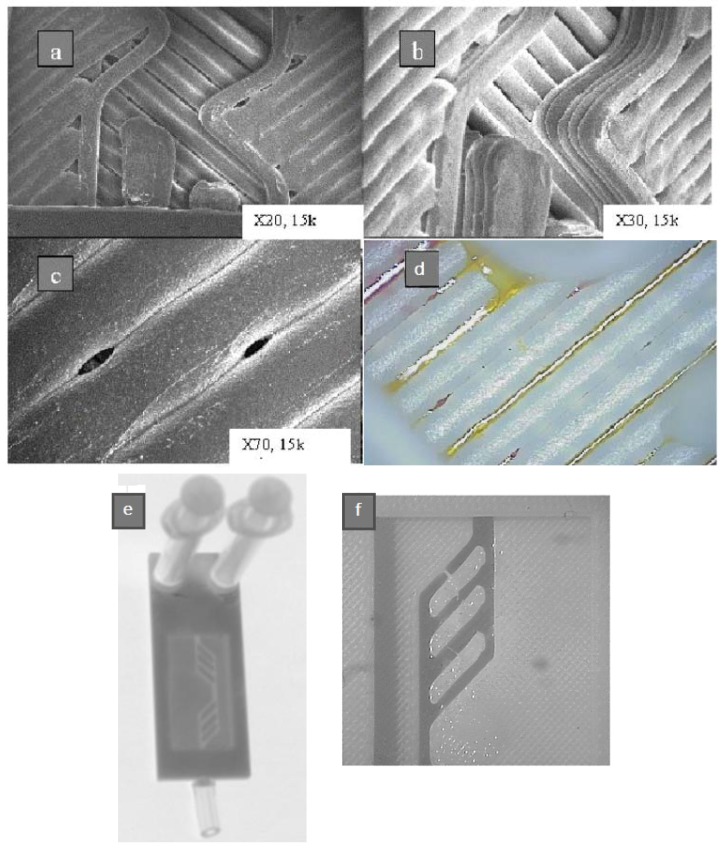
(**a**–**c**) Microchannels printed with fused filament fabrication (FFF) 3000 and their surface at different magnifications; (**d**) fluid left in the porous structure of channels; (**e**) microfluidic device realized with FFF technology; (**f**) close-up view of the channels. (Reproduced with permission from [37].)

The first examples of direct printing of microfluidic devices highlighted the strong limitations of FFF due to inaccuracy of the machines and gaps between the printed paths (see Figure 3), which yield structures unable to confine fluids that consequently suffered leakage [37]. Cavities and debris left in the structure also led to nonuniform flow.

**Figure 4:**
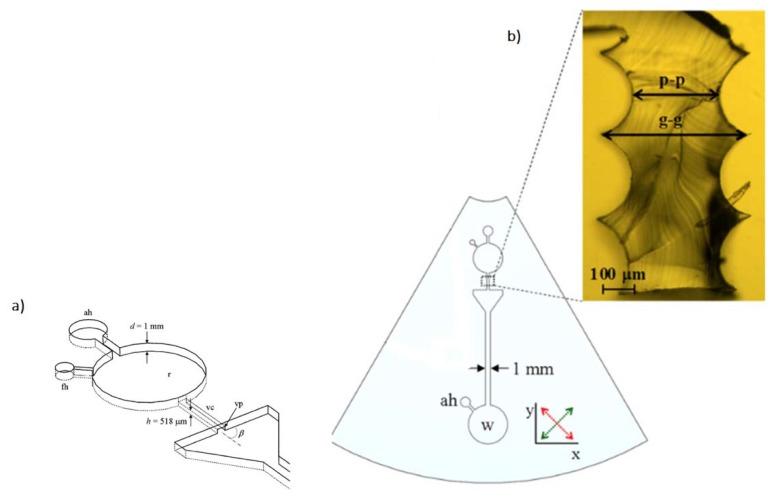
(**a**) Microfluidic architecture and tunnel structures. “ah” is air hole, “fh” is fill hole, “r” is the test fluid reservoir, “vc” is the valve channel, “vp” is the valve point or point of rapid channel expansion, “d” is the feature depth, and “h” is the valve channel height; (**b**) Microfluidic architecture showing a detail of the valve channel cast in PDMS (size 254 × 1016 μm^2^). “p-p” is the measured peak-to-peak distance, and “g-g” represents the groove-to-groove distance. (Reproduced with permission from [25].)

These aspects defined the main technology challenges and have been addressed in later work. One of the first microfluidic complete applications, not surprisingly built on well-established lab-on-a-disk principles [41], simplified the manufacturing process [25]. Such work focused its attention on capillary valves, a central element in lab-on-a-disk devices, and entirely produced such elements with FFF (Figure 4).

Modular reactors with embedded functional features have also been demonstrated with FFF, in the so-called millifluidics and reactionware devices that were the central focus of the work by Cronin and co-workers [42].

**Figure 5:**
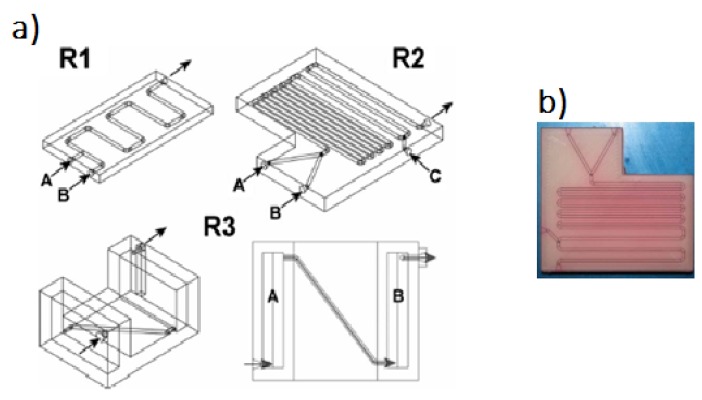
(**a**) CAD drawings of three devices: R1: Two-inlet device. R2: Three-inlet device. R3: Oblique and aerial view of a one-inlet device with two “silos,” one filled with sodium molybdate (A) and the other filled with hydrazine dihydrochloride (B); (**b**) Three-inlet device printed. In order to make its channels visible, they were filled with a methanol solution of rhodamine B dye (completely removable with washing). (Reproduced with permission from [17].)

Several configurations of reactionware devices for chemical syntheses can be manufactured in a few hours, thus producing reliable and robust reactors at low costs and utilizing well-defined materials, a main advantage of FFF in comparison to SLA technologies [17]. Two-, three-, and one-inlet mixing devices and a one-inlet device with two reservoirs, which allows the introduction of reactants during the fabrication process, are shown in Figure 5.

By using the 3D printer to initiate chemical reactions and printing the reagents directly into a 3D reactionware matrix, a more systematic manufacturing workflow was achieved [30]. Within this context, the processing advantages of flow chemistry for the synthesis of organic compounds was demonstrated [32].

**Figure 6:**
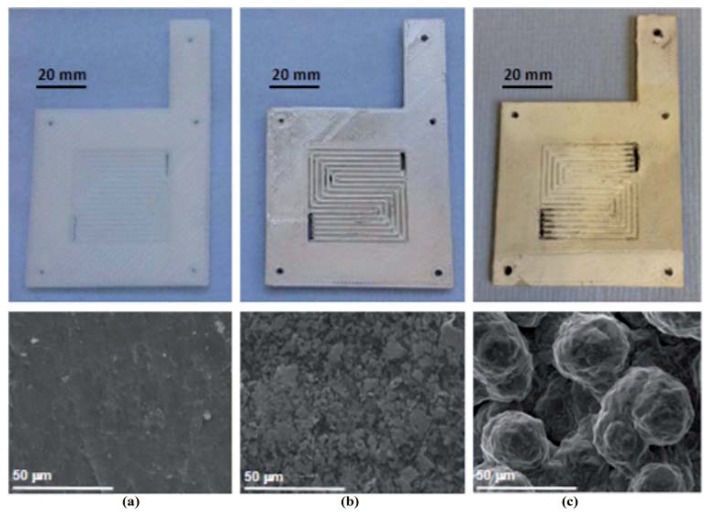
(Chisholm et al.) Photographs and relevant scanning electron microscopy (SEM) images of polypropylene (PP) flow plates. (**a**) Uncoated PP; (**b**) Curing of the second coat of silver paint; (**c**) Electrodeposition of silver. (Reproduced with permission from [26].)

Proton exchange membrane electrolyzers, made of silver-coated 3D printed components, have also been shown using PP-fabricated parts (Figure 6). The authors claimed an excellent performance for a first-generation device in terms of overall efficiency, internal resistances, and current–voltage response.

### 4.2. Devices Based on 2D Closed Channels

**Figure 7:**
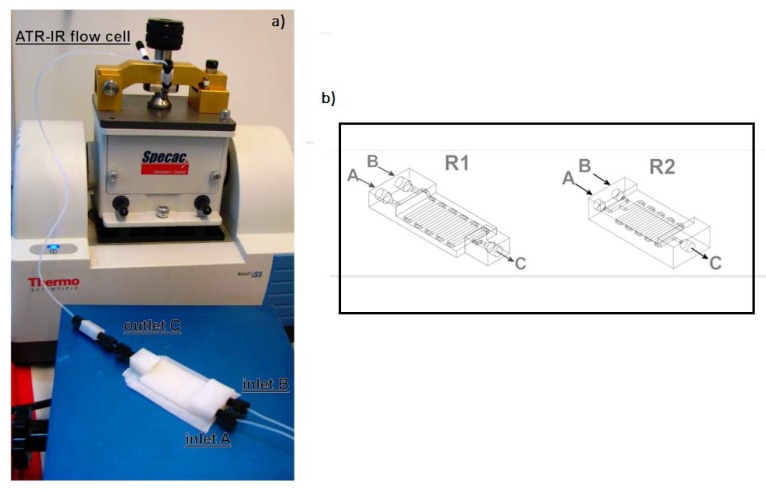
(Dragone et al.) (**a**) Flow system setup; (**b**) Schematic representation of the 3D-printed reactionware devices with two inputs and one output, showing the internal channels. The dimensions of the inlets/outlets are 3 mm in R1 and 6 mm in R2. (Reproduced with permission from [32].)

Additively manufactured reactionware devices (Figure 7) were connected with standard fittings, thus resulting in versatile, custom-made modular fluidic systems. Serial reactors with in-line, real-time analysis were included [32]. Two types of organic reactions, imine syntheses and imine reductions, were used to demonstrate how two different functional substrates yield different products.

**Figure 8:**
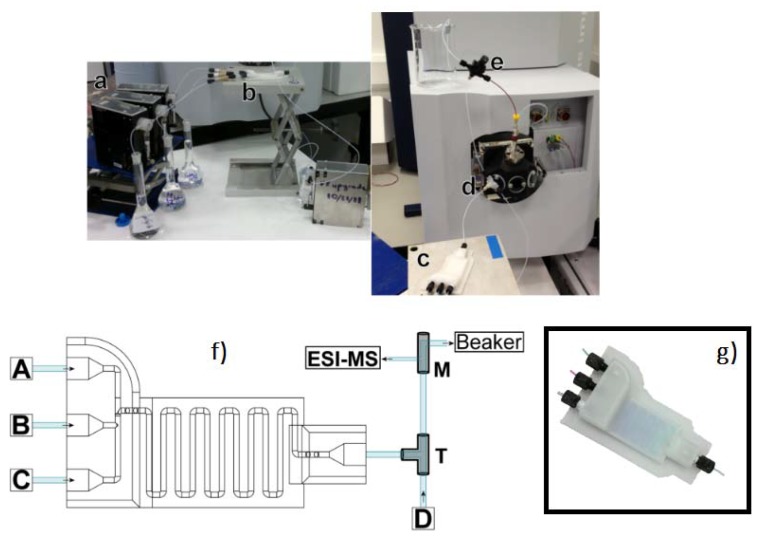
Device setup and connection to the mass spectrometer. Three inlets are connected to a syringe pump; the outlet is directly connected to a T-piece, where it mixes with a stream of MeOH for dilution. A Polyetheretherketone (PEEK) microsplitter valve is used to split the stream so that only a part of the flow-rate reaches the ESI-MS. (**a**) = syringe pumps; (**b**) = screw fittings; **c** = 3D-printed device; (**d**) = T-piece; (**e**) = PEEK microsplitter valve; (**f**) schematic view of the device setup (**g**) actual device with screw fittings and connected with 1/16 inch (1.6 mm) tubing. (Reproduced with permission from [18].) The devices were treated with dichloromethane (DCM) vapor for up to 15 min and 1 min to create a smooth and soft surface finish, which were then exposed to an oxygen plasma to bond with flexible silicone-based polymer, to create soft surfaces for inter-connectable modular parts. As highlighted by the authors, there is an advantage of using FFF-based 3D printers instead of stereolithography (SLA)-based printers, because the printing materials for SLA-based printers are mostly proprietary and their chemical compositions are unknown, whereas those for FFF-based printers are well described. (Reproduced with permission from [18].)

Figure 8 illustrates an application of such a FFF tailored made reactor, in this case directly linked to a high-resolution electrospray ionization mass spectrometer for real-time, in-line observations [18].

Here, 3D-printed cartridges for paper spray ionization were demonstrated in connection to reservoirs for solvent supply, thus allowing prolonged spray generation from a paper tip [21]. The paper provides capillary action for transport from the PLA cartridge manufactured by FFF.

Mixers, aimed at biomolecular applications, have also been configured with FFF platforms [22]. A sandwich-format design was proposed to allow the implementation of multiple spectroscopic probes in the same mixer. The channels were defined by void regions in the polymer acting as spectral windows in the region of interest.

**Figure 9:**
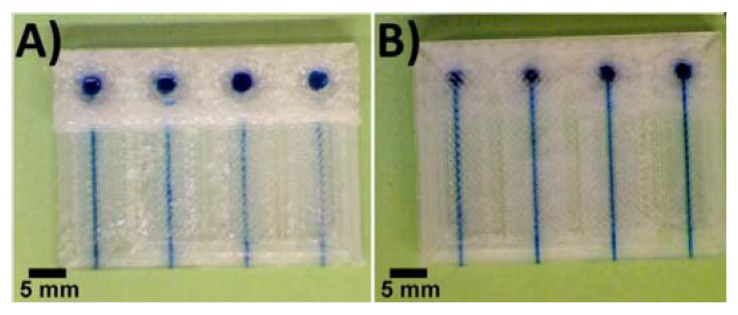
Top view (**A**) and bottom view (**B**) of semi-transparent 3D-printed 800 μm × 800 μm square cross-sectional PET channels filled with 1 mM methylene blue solution. (Reproduced with permission from [33].)

**Figure 10:**
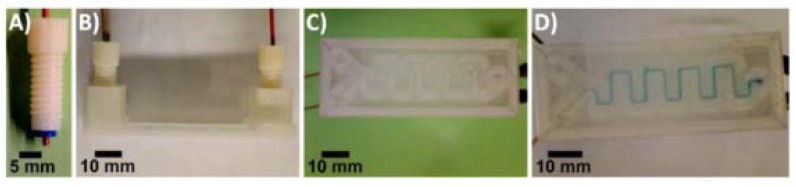
Preparation of Prussian blue nanoparticles (PBNPs) using a 3D-printed Y-shaped mixing device. (**A**) D-printed ABS fitting (**B**) side view of the device (**C**) bottom view of the device (**D**) bottom view of the device after mixing 5 mM iron (II) chloride and 5 mM potassium ferricyanide solutions to form citrate-PBNP. (Reproduced with permission from [33].)

**Figure 11:**
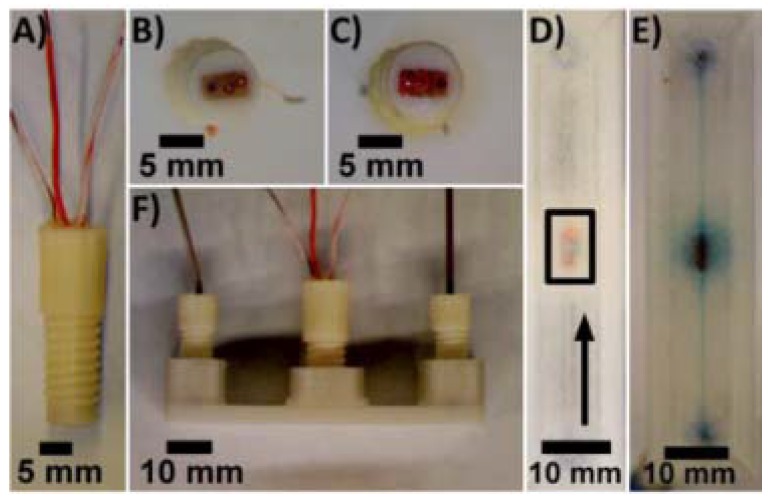
Electrodes embedded in the fluidic channel. (**A**) Threaded ABS fitting with integrated electrodes; (**B**,**C**) Bottom view of the fittings equipped with different reference, working, and counter electrodes; (**D**) Bottom view of a 3D-printed PET device with reference, working, and counter electrodes (bottom to top) incorporated in the fluidic channel. The arrow indicates direction of flow. (**E**,**F**) Bottom and side views, respectively, of the device filled with methylene blue in PBS. (Reproduced with permission from [33].)

Consumer-grade FFF machines have also been used to fabricate low-cost fluidic devices, replacing previous versions implemented with more expensive AM methods [33]. These include nanoparticle preparation and electrochemical sensing (Figure 9), with devices manufactured in PET, connectors in ABS and tubes in Polyetheretherketone (PEEK), see Figure 10 and Figure 11. The channels have been designed to have 800 × 800 µm^2^ square cross-sections and are semi-transparent to allow visualization of the solution-filled channels, owing to the PET.

**Figure 12:**
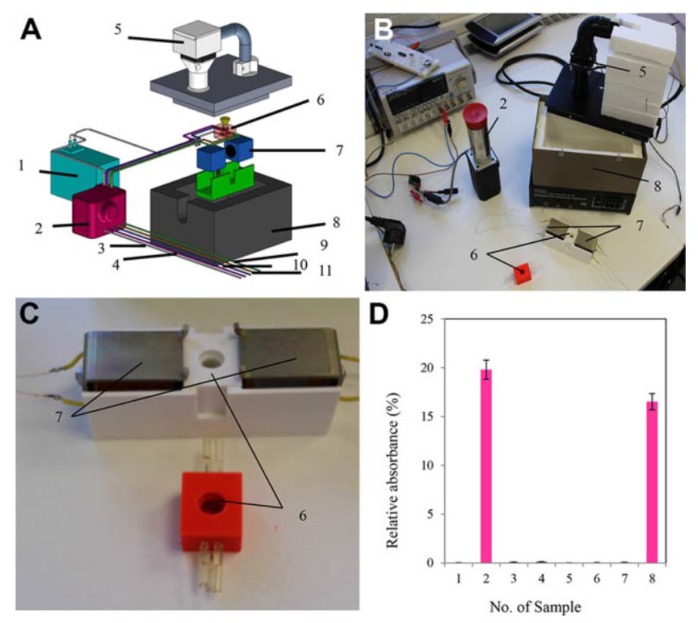
(**A**) Scheme of 3D-printed chip for detection and confirmation of MRSA presence using binding of MRSA to the gold nanoparticles with specific primers in the chip; (**B**) system for the identification of MRSA in the sample; and (**C**) reaction chamber of 3D-printed chip: 1—spectrophotometric detector, 2—pump with the valves, 3—outlet, 4—the first inlet hose, 5—thermoregulatory system, 6—cultivation chip, 7—electromagnet, 8—thermoisolating box, 9—the second inlet hose, 10—the third inlet hose, and 11—the fourth inlet hose; (**D**) Comparison of reaction mixtures relative absorbance obtained from 3D-printed chip: various PCR products of bacterial strains: (1) *S. aureus*, (2) MRSA, (3) *E. coli*, (4) *S. typhimurium*, (5) *L. rhamnosus*, and three clinical specimens, where the presence of *S. aureus* was confirmed (sample numbers 6–8). The results are expressed as percentage of the AuNPs signal (100%).” (Reproduced with permission from [34].)

In Figure 12, bacterial cultivation, DNA isolation, polymerase chain reaction, and detection of amplified gene sequences of methicillin-resistant Staphylococcus aureus (MRSA) were also implemented in FFF devices [34]. The implementation of this rapid and inexpensive diagnostic system with high sensitivity and specificity is very important for the prevention of resistant bacteria to become an emerging public health threat.

**Figure 13:**
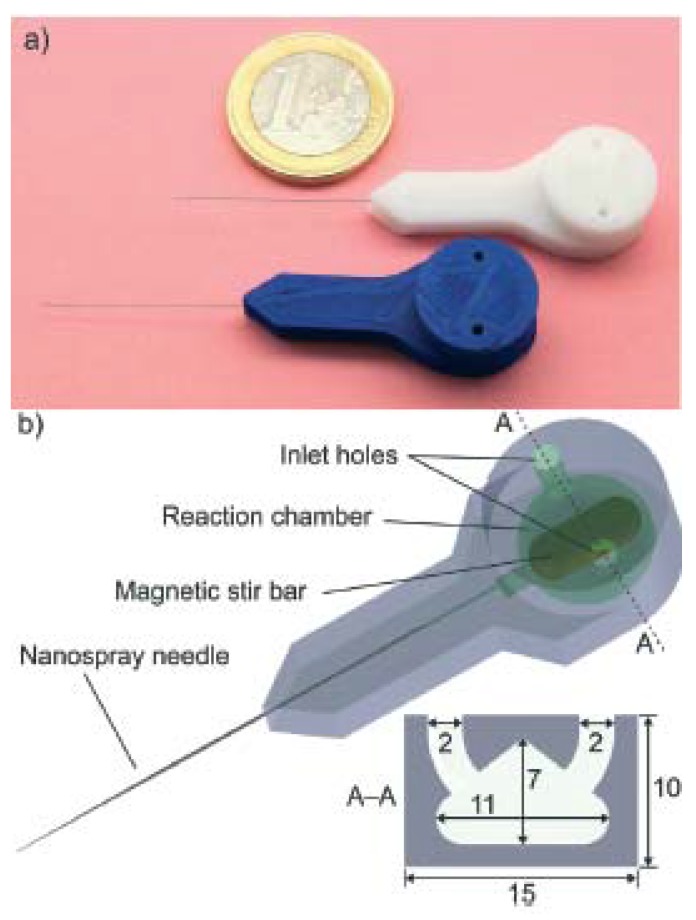
(**a**) 3D printed reactors from white and blue PP. PP weak mechanical properties and poor adhesion to most built platforms are compensated by the fact that it is inert and resistant to many inorganic and organic reagents and solvents; (**b**) functional parts and cross-section of the reaction chamber, dimensions in millimeters. (Reproduced with permission from [19].)

**Figure 14:**
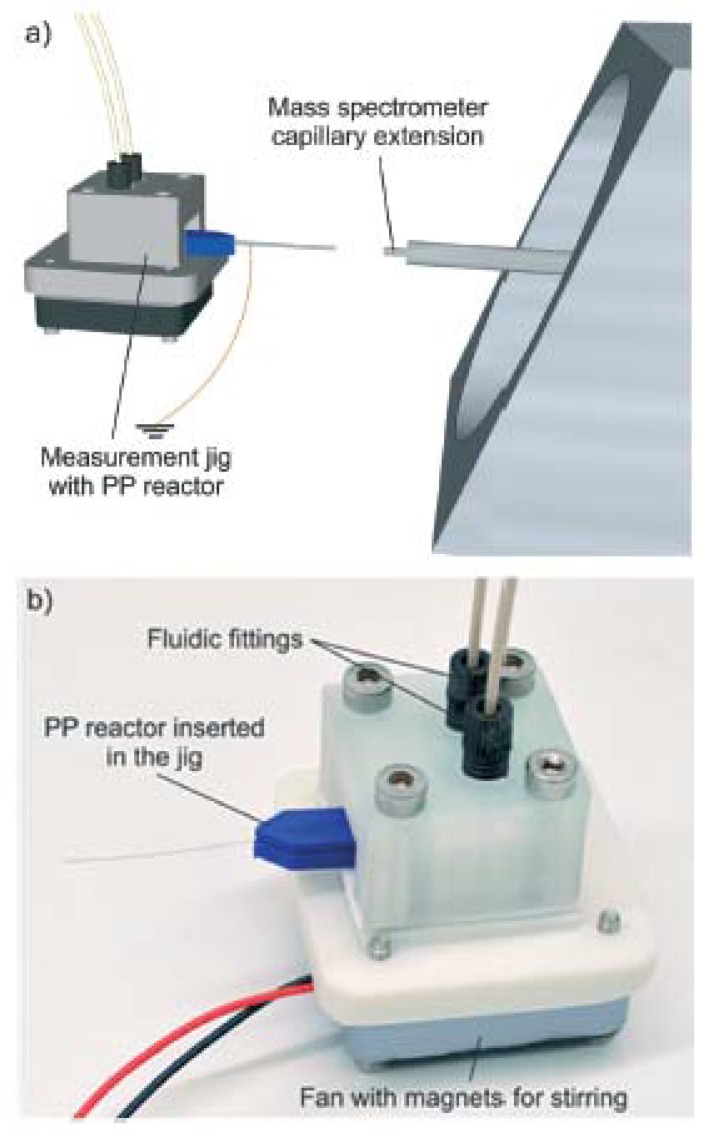
(**a**) Measurement setup; (**b**) Measurement jig with a miniaturized reactor inserted. (Reproduced with permission from [19].)

Using the FFF printer miniFactory 3 by miniFactory Oy Ltd., equipped with a 0.4-mm diameter nozzle, a miniaturized PP reactor was fabricated in order to implement very fast mass spectrometric chemical reaction monitoring [19]. During the 3D printing process, a stainless steel nanoelectrospray ionization capillary and a magnetic stir bar were embedded into the PP reactor, allowing both the ionization of the analytes and the direct sampling of a reaction solution without external pumping (see Figure 13 and Figure 14). The benefits of this solution include: minimal dead volume, direct sampling of the reaction solution without external pumping (the electrospray process automatically pulls liquid from the reaction chamber via the nano-ESI capillary), and very fast reaction monitoring thanks to the minimization of the volume between the reaction chamber and the ion source.

### 4.3. Devices Based on 3D Geometry

Recently, a general approach for manufacturing chemical reactors, termed reactionware, using extrusion-based 3D printing has been presented [12]. The proposed procedure describes the printing of a PP architecture concurrently with soft material catalyst composites, exploiting the FFF capability of extruding more than one material on the same slice. The protocol then further describes the preparation of composite catalyst–silicone materials for incorporation into the 3D-printed device and the steps required to fabricate a reactionware device. The approach aims for versatility in the design and use of reactors customized by the experimental user. The procedure results in the production of a sealed reactor for multistep organic synthesis. The authors claim a possible duration of the whole process of 3 days.

**Figure 15:**
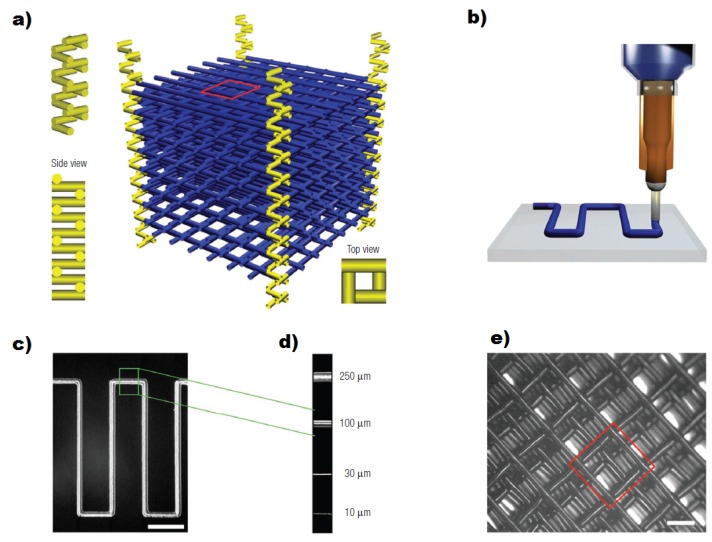

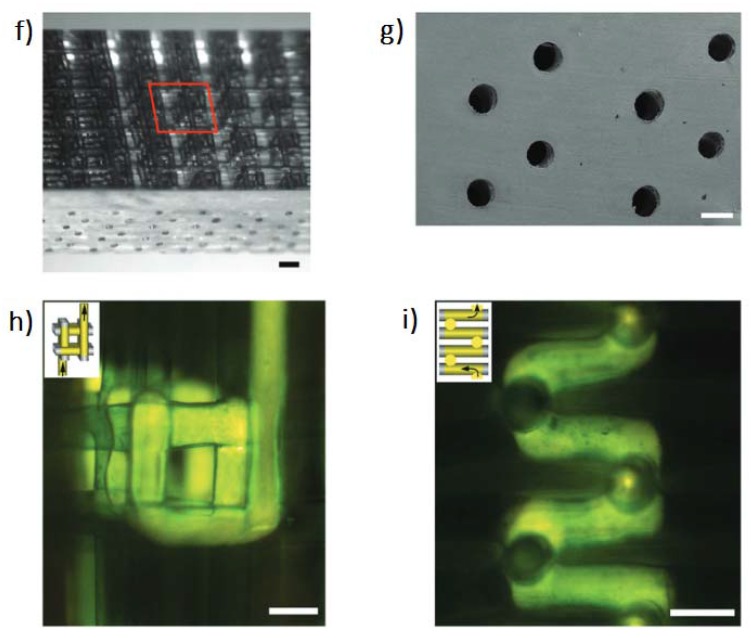
Microvascular scaffold fabrication. (**a**) Square-spiral tower structure (yellow) embedded within the 3D microvascular network (blue); (**b**) Robotic deposition of the fugitive organic ink (blue) through a cylindrical nozzle onto a moving x–y stage; (**c**) Optical image (top view) of a 2D patterned feature within the microvascular scaffold; (**d**) Ink filaments produced from different nozzle diameters; (**e**) Optical image (top view) of microvascular scaffold; (**f**) 3D microvascular network embedded in clear epoxy matrix. Scale bar = 0.50 mm; (**g**) Scanning electron microscope image of the cross-section of this network. Scale bar = 250 μm; (**h**–**i**) Fluorescent microscope images (top view and side view respectively) of green fluid flowing inside a single tower structure showing its isolated nature. Scale bars = 250 μm. (Reproduced with permission from [43]).

Therriault et al. [43] used sacrificial FFF printing to create a complex 3D scaffold of cylindrical segments using paraffin-based organic ink and subsequently embedded the scaffold with a UV-curable epoxy resin. Using this technique, sixteen-layer scaffolds were produced by deposition in a layerwise build sequence. Heating to 60 °C, the organic ink was thermally removed under a light vacuum, leaving the epoxy hollow geometry behind, thus fabricating 3D microvascular networks for microfluidic blood flow (Figure 15).

**Figure 16:**
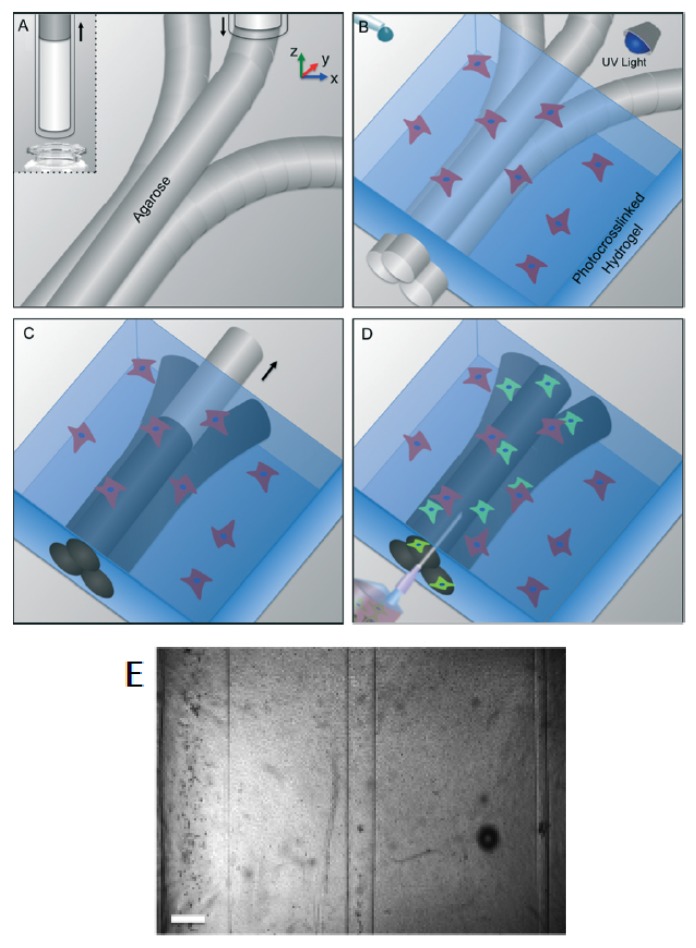
Bioprinting of agarose template fibres and microchannels fabrication with template micromoulding. (**a**) The agarose is aspirated by a bioprinter. After a 4 °C thermal stage, agarose fibers are bioprinted at predefined locations; (**b**) A hydrogel precursor is casted over the bioprinted mould and photocrosslinked; (**c**) The template is removed from the surrounding photocrosslinked gel; (**d**) Formation of fully perfusable microchannels (**e**) microchannels representation (from left to right: 1000 μm, 500 μm, and 150 μm diameter, 500 μm scale bar). (Reproduced with permission from [44].)

Another example of indirect manufacturing is shown in Figure 16 [44]. In this case, a 3D moulding technique employs bioprinted agarose fibers to fabricate microchannel networks with various architectural features within photocrosslinkable hydrogel constructs, thus creating a synthetic vascularization and physiological micro-environment for organ-on-a-chip studies.

When using photocrosslinkable hydrogels, the path length for UV light exposure increases with the size of the construct, causing variable crosslinking properties throughout the thickness of the gel and limiting the effective size of the constructs. However, this limitation can be overcome using interlocking microstructural features in microfabricated microgels, as the authors suggest.

**Figure 17:**
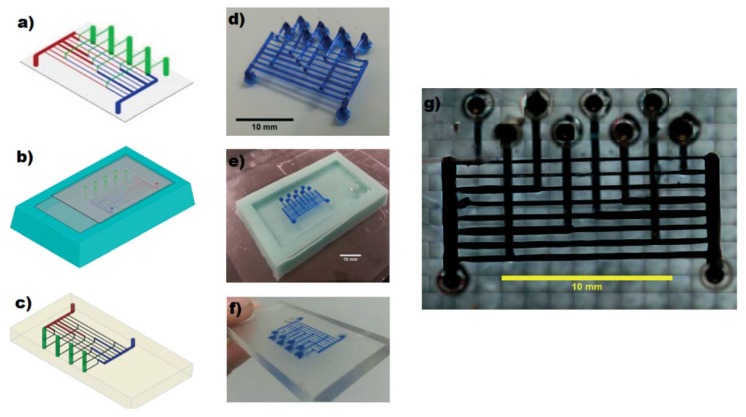
Gelber and Bhargava Microfluidic devices produced by moulding features printed with FFF technique. (**a**–**c**) Generator design; (**d**) FFF printed sacrificial isomalt scaffold; (**e**) Embedding of the scaffold in agarose for casting; (**f**) The carbohydrate quickly dissolves in the agarose hydrogel; (**g**) Filling of the scaffold replica with black dye. (Reproduced with permission from [45].)

Sacrificial lattices of isomalt were also employed to create dilutors [45], where the sugar was cast in an agarose mould and dissolved into the hydrogel in few minutes, in order to create a network (Figure 17); this was tested and visualized after being perfused with a colored solution.

**Figure 18:**
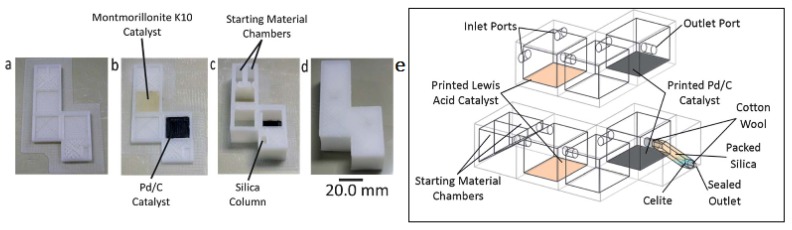
(**a**) Reactor base with purification column before printing of catalyst regions; (**b**) Reactor base with purification column after printing of catalyst regions; (**c**) Fabricated reactor with purification column after addition of starting materials, reagents, and packing of silica; (**d**) Final sealed reactor; (**e**) Schematic diagram of the 3D-printed sequential reactors. (Reproduced with permission from [31].)

The combination of modular units produced with two 3D-FFF machines with extruders for solid and liquid materials have also been used to produce pluggable reactionware integrated into monolithic devices [31]. The device was operated in rotating mode, using gravity to drive the flows through successive stages, and avoiding any pumps or more advanced liquid-handling (Figure 18).

FFF array reactors were also used to synthesize two new coordination polymers, optimize the synthesis of one of these, and scale up its synthesis using larger reactors produced with the same 3D printer [27]. The reactors were designed with hollow chambers in the interior of each device surrounded by PP walls of thickness 4 mm. The filling of the reagents was performed after pausing the fabrication at 80% of completion. Resuming the printing yielded a hermetically sealed, monolithic structure, containing the reaction mixture.

**Figure 19:**
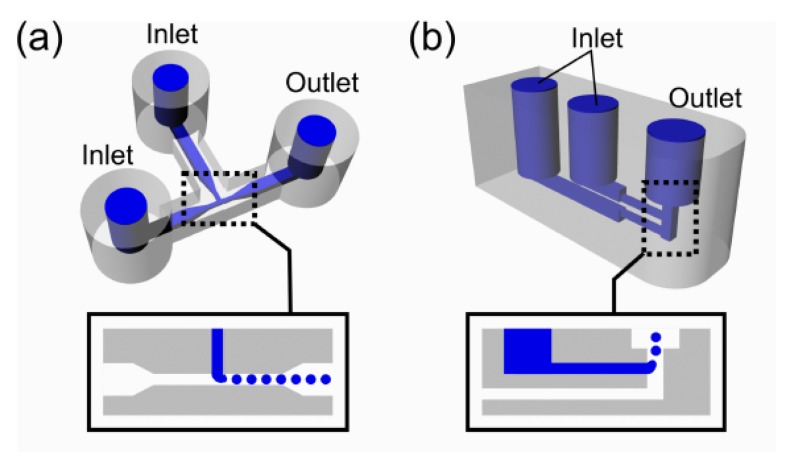
3D printed droplet generators. (**a**) T-junction device with one aqueous inputs, one oil input, and one output. The aqueous phase was introduced from the top inlet and oil phase from the left; (**b**) The aqueous phase was cut into small droplets by oil phase. (Reproduced with permission from [24].)

**Figure 20:**
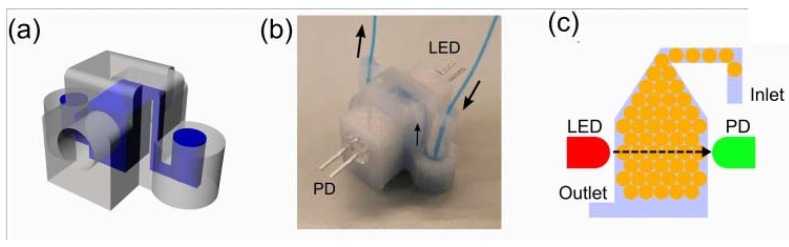
(**a**) Droplet storage and growth measurement device CAD design; (**b**) Fabricated device with LED and photodiode (PD) sensor attached; (**c**) Scheme of droplet cell growth monitoring. (Reproduced with permission from [24].)

**Figure 21:**
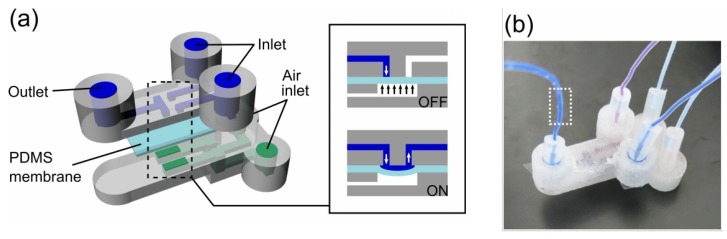
(**a**) CAD design of flow selector device using PDMS membrane valve. Valves are closed when air pressure is applied from the bottom channel. When opened, aqueous phases pumped at a constant pressure flow through the valve; (**b**) 3D-printed flow selector device. (Reproduced with permission from [24].)

**Figure 22:**
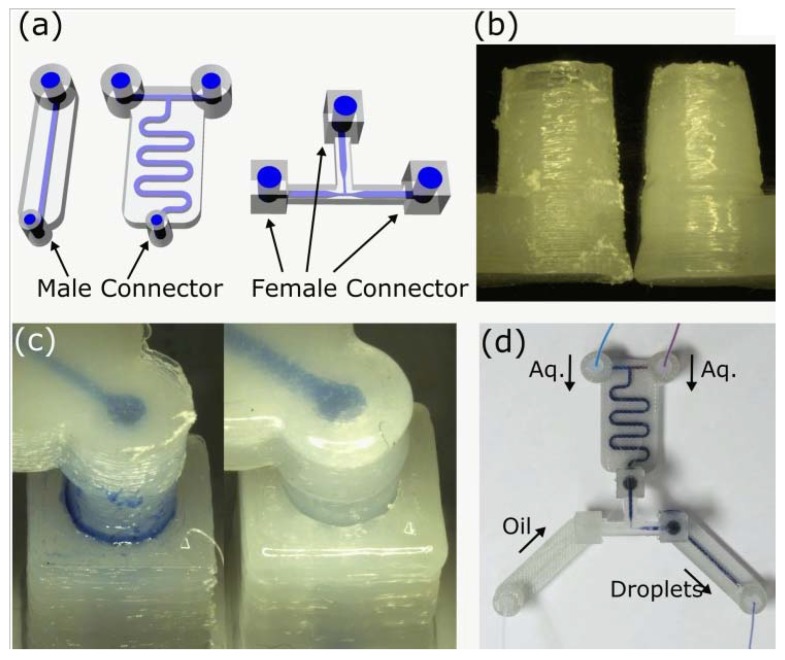
(**a**) Modularised 3D-printed device CAD designs; (**b**) Surface roughness differences between untreated male connector (left) and vapor-treated connector (right). (**c**) Close view of connected male/female parts; (**d**) Assembled modular devices. (Reproduced with permission from [24].)

Additively manufactured droplet generators for programmable liquid handling and control of biological samples were also demonstrated [24]. Mono-disperse droplets of water-in-oil (W/O) were generated, which included a sensor-integrated chamber for online monitoring of cellular growth. Moreover, chemical surface treatments were introduced to allow valve-based flow selector for liquid flow control and inter-connectable modular parts for networking fluidic parts (Figure 19, Figure 20, Figure 21 and Figure 22).

**Figure 23:**
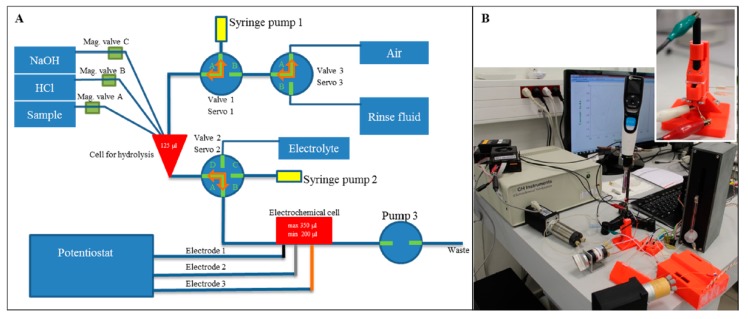
Vlachova et al. microfluidic system: (**A**) microfluidic system scheme composed of hydrolysis and detection parts; (**B**) microfluidic system and detailed picture of the chip with the electrodes. (Reproduced with permission from [35].)

ABS chips have also been developed (Figure 23). Such devices included one chamber for the hydrolysis of the nucleic acid and another for the electrochemical detection by modified glassy carbon electrode [35]. This module allows the replacement of the electrode, whereas complementary parts of the chip provided for electrochemical detection. The main parts of the detection unit, including the holder, detection cell, and pressure cover, were made of ABS. The whole construct was completed by two 3D sealing parts realized with a flexible material (Elastic Printplus Natural from 3DFactories).

**Figure 24:**
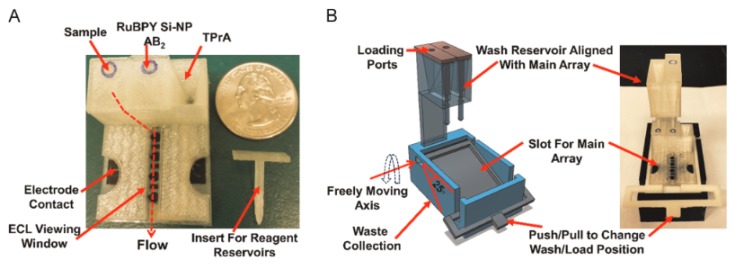
(**A**) Basic 3D-printed array showing three reagent reservoirs equipped with inserts and flow path for reagents; (**B**) Wash reservoir module (left) and assembled immunoarray setup with both main array and wash module (right). (Reproduced with permission from [23].)

**Figure 25:**
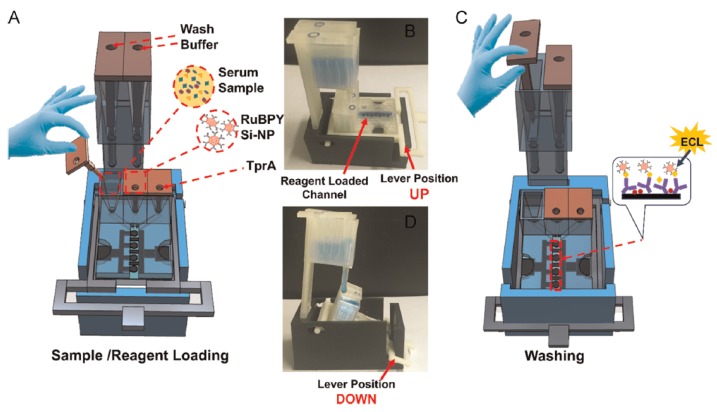
FFF-made immunosensor device [23]. A: Removal of insert for sample delivery from reservoir by gravity flow. B: Blue-color solution fills the horizontal detection channel with lever up. C: Buffer delivery from wash reservoir to detection channel in order to wash away unbound proteins. D: Blue-color solution is delivered from wash reservoir to main array when lever is down. (Reproduced with permission from [23].)

Immunosensors to detect three cancer biomarker proteins in serum were also demonstrated (Figure 24 and Figure 25). Low-cost screen-printed carbon sensors with gravity flow for sample/reagent delivery and washing was implemented [23], including prostate-specific antigen (PSA), prostate-specific membrane antigen (PSMA), and platelet factor-4 (PF-4) in serum. The platform aimed at sensitive on-site cancer diagnostic at very low system cost, owing to AM.

## 5. Discussion

In the last five years, the evolution of Filament Freeform Fabrication, especially in the professional grade segment, has been mainly driven by companies, rather than academic world, with the introduction of new technological solutions mainly oriented to improve the positioning accuracy and improve the extrusion process for better performing materials and for smaller extrusion diameters. As regards positioning accuracy, stepper motors have reached a high enough level of performance to make the cinematic transmission the most critical aspect. In fact, state of the art professional 3D printers, exploit rubber belts being subject to distortion, wear, motion repeatability, and requiring continuous calibrations. As an example, the use of racks and helical pinions in tempered steel has been proposed by Roboze^TM^, who has also worked in the field of extruding high-performing polymers such as polyether ether ketone (PEEK). The solution proposed is actually the state of the art in the professional segment, consisting in an internal channel of the extruder that accelerates high viscosity polymers during the extrusion process to decrease viscosity at high temperatures, controlling the die-swelling phenomenon. One of the main limitations of the FFF process for microfluidics and microfabrication, in general, is the minimum slice height and the bead (sometimes called road) width on the XY plane, which is correlated to the nozzle diameter and to the extrudability of the material. Small slice heights and bead diameters allow the fabrication of smaller details and are feasible when small nozzle diameters are exploited, in conjunction with materials capable of flowing into such nozzles without obstructions. Another relevant limitation of FFF printers, when compared to their closest competitors, the SLA printers, is the sequential character of FFF printers, and the limitations to accelerate the material deposition. SLA printers can rapidly scan a laser covering each printed layer in seconds, whereas for that same area, an FFF printer can take much longer owing to the necessity to extrude the materials. In addition, DLP-SLA printers simultaneously expose each layer of photo-curable resin at the resolution of the digital micro mirror projector (e.g., HD: 1920 × 1080 pixels). To match this parallel exposure would require an impractical similar number of FFF extrusion heads.

Regarding the z-axis resolution, in both SLA and FFF technologies, longer fabrication times are required for a gain in resolution that is mostly in large AM objects, rather than those in microfluidics manufacturing. Microfluidic devices are normally printed without bases or supporting structures and are aligned to the printer head or perpendicular to it.

### 5.1. Comparison with Photopolimerization Processes

SLA and FFF are the most common technologies in microfluidics and in several other applications, while Polyjet is particularly promising for microfabrication owing to its inherent manufacturing accuracy. In this context, in [46], the dimensional measurements of a micromixer, fabricated in four samples, are reported using the following machines for the corresponding processes: (i) stereolithographic Formlabs Form 2; (ii) FDM Stratasys F370; (iii) FFF Ultimaker 3; and (iv) Stratasys Objet 30 (Polyjet process). The micromixer consisted of two inlets, one outlet, and an 18-channel serpentine, which is able to mix two fluids in a laminar flow to achieve mixing by diffusion.

The 3D models for the micro-mixers realized through SLA, FFF and Polyjet present very low and similar differences between measured and nominal values of the average channel depth, lower than 20 μm. In [47] an experimental comparison of three 3D printing technologies is conducted using a very similar Y-junction microfluidic device. In this case the processes compared are: fused deposition molding (FDM), Polyjet, and digital light processing stereolithography (DLP-SLA). An evaluation of the printer performance is reported in terms of feature size, accuracy, and suitability for mass manufacturing, monitoring the laminar flow. FDM was declared suitable for microfabrication with minimum features of 321 ± 5 μm, and rough surfaces of 10.97 μm, indicating a strength in fabricating micromixers. Polyjet fabricated channels with a minimum size of 205 ± 13 μm, and a surface roughness of 0.99 μm. The DLP-SLA fabricated a minimum channel size of 154 ± 10 μm, and 94 ± 7 μm for positive structures such as soft lithography templates, with a roughness of 0.35 μm. The authors classified the three 3D printing technologies in this way: FDM is the most suitable for mixing, due to high roughness; Polyjet printing is more suited for microfluidic applications where flow splitting is not required, such as cell culture or droplet generators and DLP-SLA and microfluidic applications requiring precise control of flow.

### 5.2. Safety

Sometime called “Desktop Manufacturing,” FFF is widely known as one of the safest additive processes due to several factors: (a) no monomers or unstable chemical substances are employed, (b) no powders are employed, and (c) biocompatible and intrinsically safe materials can be extruded, hence with very low hazards. Consequently, the risk is mainly related to the material extruded, as it is described in the safety data sheet. On the other hand, the need of chemical post treatments can expose the operators to hazards, using solvents. However, it must be considered that PLA is rarely treated with solvents, and ABS is the most treated material, with Acetone needing limited precautions.

Moreover, the improvement in FFF machines, in positioning accuracy and deposition strategy, plus the ironing function recently introduced on open source slicing software such as Cura, will completely exclude the need of post-treatments. In fact, as reported in Table 1, most research papers do not report post treatments, especially in recent years.

## 6. Conclusions

Despite the challenges ahead, current 3D printing techniques allow multiple design interactions per day rather than per month, at affordable costs and with the fabrication skills standardized and transferred to the 3D printing systems. The FFF applications in microfluidics will be increasing, mainly owing to the high user compliancy of this process compared to other additive technologies. Other very interesting technologies in the field are stereolithography (SLA) and polyjet, mainly owing to their higher accuracy and structural density, compared to FFF. In particular, SLA is available in machines as cheap as professional grade FFFs. However, the latter technique has the advantages of simplicity and a variety of materials. The path to widen microfluidic applications passes through a reduction in the diameter of the extrusion nozzle. Standard nozzle sizes reach 200 μm, and in fact, 100 μm nozzles are also available on some machines, but the issues related to incorrect material flow inside the nozzle have not yet been overcome. Perhaps the intrinsic benefits and limitations of SLA and FFF technologies will see them solved by a combination of these technologies, exploiting the accuracy of SLA and the versatile materials choice of FFF.

AM has gained importance in the microfluidics community very recently, and PDMS and soft-lithography is still the method of choice. Regarding FFF adoption in the microfluidics community, it offers the highly appreciated benefit of operating with well-defined materials, which is the main drawback of SLA technologies. The compromise between resolution and material choice will certainly continue to define the preference for either technology.

## Figures and Tables

**Table 1:** Summary of the microfluidic applications of FFF related to machine specifications.

Machine	Paper	Material	Method	Treatment	Channel Depth × Width [µm] Diameter (D) in Case of Circular Channel Radius (R) in Case of Semicircular Channel	Declared Minimum Slice Height (μm)	Ref. Figure
Airwolf3D HD2	Kise et al., 2015 [22]	PLA	Direct	Annealing at 170 °C	D (406 ± 38.9) before annealing D (151 ± 29.1) after annealing	60	-
Bits from Bytes 3D Touch	Kitson et al., 2012 [17]	PP	Direct	No treatments described	R 400	125	Figure 5
Bits from Bytes 3D Touch	Dragone et al., 2013 [32]	PP	Direct	No treatments described	D 1500	125	Figure 7
Bits from Bytes 3D Touch	Mathieson et al., 2013 [18]	PP	Direct	No treatments described	D 1500	125	Figure 8
Bits from Bytes 3D Touch	Chisholm et al.,2014 [26]	PP	Direct	PP with two coats of silver paint applied subsequently, and then cured at 120 °C for electrocoating.	900 × 900	125	Figure 6
Bits from Bytes 3D Touch	Kitson et al., 2014 [27]	PP	Direct	Silver coating and curing 120 °C	900 × 900	125	-
Fab@Home platform	Symes et al., 2012 [30]	Acetoxy silicone polymer (Loctite 5366 bathroom sealant, LOCTITE) with inserts of nonprintable materials	Direct	No treatments described	n.d.	n. d.	-
Fab@Home Version 0.24 RC6 freeform fabricator+ Bits from Bytes 3D Touch	Kitson et al., 2013 [31]	PP basis and active reagents into an acetoxy silicone polymer matrix	Direct	No treatments described	n.d.	n. d.	Figure 18
Felix 3D Printer	Salentijn et al., 2014 [21]	PLA	Direct	Isopropanol exposition to improve fast initial wetting	n.d.	170	-
Leapfrog Creator	Thompson, 2014 [36]	PLA (support) and ABS	Direct	Sonicating for support removal	n.d.	20	-
Makerbot Replicator 2	Bishop et al., 2015 [33]	PET and ABS	Direct	No treatments described	800 × 800	100	Figures 9–11
Makerbot Replicator 2	Kadimisetty et al., 2016 [23]	PLA	Direct	No treatments described	n.d.	100	Figures 24 and 25
MiniFactory 3	Scotti et al., 2017 [19]	PP	Direct	No treatments described	D 2000	20	Figure 13
Profi3Dmaker	Chudobova et al., 2015 [34]	ABS	Direct	No treatments described	n.d.	80	-
Profi3Dmaker	Vlachova et al., 2015 [35]	ABS	Direct	No treatments described	n.d.	80	Figure 23
Stratasys Dimension	Moore et al., 2011 [25]	ABS	Direct	Sanding	R 500	254	Figure 4
Stratasys Dimension	McCullough & Yadavalli, 2013 [20]	ABS	Direct	Acetone-based sealing method, described as minimally impacting on surface roughness and structural fidelity; subsequently a photo-induced graft polymerization of poly (ethylene glycol) functionalities	1000 × 500	254	-
Stratasys FFF 3000	Hengzi et al., 2001 [37]	ABS	Direct	No treatments described	500 × 500	178	Figure 3
Ultimaker 2	Tsuda et al., 2015 [24]	PLA and PET	Direct	Dichloromethane (DCM) vapor for up to 15 min and 1 min	400 × 400	20	Figures 19–22
Zhejiang Flashforge 3D Technology Co.	He et al., 2015 [38]	Fused sugar powder	Indirect	No treatments described	D 200	100	-
